# High-resolution gridded population datasets for Latin America and the Caribbean in 2010, 2015, and 2020

**DOI:** 10.1038/sdata.2015.45

**Published:** 2015-09-01

**Authors:** Alessandro Sorichetta, Graeme M. Hornby, Forrest R. Stevens, Andrea E. Gaughan, Catherine Linard, Andrew J. Tatem

**Affiliations:** 1 Geography and Environment, University of Southampton, Highfield Campus, Southampton SO17 1BJ, UK; 2 Institute for Life Sciences, University of Southampton, Highfield Campus, Southampton SO17 1BJ, UK; 3 GeoData, University of Southampton, Highfield Campus, Southampton SO17 1BJ, UK; 4 Department of Geography and Geosciences, University of Louisville, Louisville, KY 40292, USA; 5 Lutte biologique et Ecologie spatiale (LUBIES), Université Libre de Bruxelles, CP 160/12, 50 Avenue F.D. Roosevelt, Bruxelles B-1050, Belgium; 6 Fonds National de la Recherche Scientifique, 5 rue d'Egmont, Bruxelles B-1000, Belgium; 7 Fogarty International Center, National Institutes of Health, 16 Center Drive, Bethesda, MD 20892, USA; 8 Flowminder Foundation, Roslagsgatan 17 SE-11355, Stockholm, Sweden

**Keywords:** Geography, Malaria, Sustainability, Environmental sciences

## Abstract

The Latin America and the Caribbean region is one of the most urbanized regions in the world, with a total population of around 630 million that is expected to increase by 25% by 2050. In this context, detailed and contemporary datasets accurately describing the distribution of residential population in the region are required for measuring the impacts of population growth, monitoring changes, supporting environmental and health applications, and planning interventions. To support these needs, an open access archive of high-resolution gridded population datasets was created through disaggregation of the most recent official population count data available for 28 countries located in the region. These datasets are described here along with the approach and methods used to create and validate them. For each country, population distribution datasets, having a resolution of 3 arc seconds (approximately 100 m at the equator), were produced for the population count year, as well as for 2010, 2015, and 2020. All these products are available both through the WorldPop Project website and the WorldPop Dataverse Repository.

## Background & Summary

The Latin America and the Caribbean region has a population of around 630 million and is one of the most urbanized regions in the world, with 80% of its population currently living in urban areas. Its population, which increased by 1.5 times over the last 25 years, is expected to grow by another 25% and further urbanize by 2050, with 86% living in urban areas^1^.

According to the Pan American Health Organization^2^, health and demographic indicators highlight that, although with significant variation from country to country and to a far lower degree than Africa and central Asia, the region is characterized by relatively high maternal and infant mortality rates, and a lack of access to health facilities and services for a large part of its population. In addition, many endemic infectious diseases are also present and include malaria, dengue, chikungunya, chagas, and leishmaniasis^3,4^. Furthermore, low and middle income countries located in the region are highly vulnerable to and affected by natural and man-made disasters^5^ and, according to the Fifth Assessment Report of the Intergovernmental Panel on Climate Change^6^, the frequency and intensity of weather- and water-related hazards are expected to rise in the upcoming decades, both globally and regionally, as a consequence of climate change. Finally, the rapid development of the region and its ongoing urbanization is expected to further exacerbate problems related to the rapid land-use change and deforestation in rural areas^7^ and the growth of informal settlements in urban areas^8^.

In this context, contemporary, spatially detailed, and comparable datasets that accurately depict the distribution of the residential human population are a fundamental prerequisite for measuring the impacts of population growth^9^, monitoring changes^10^, supporting environmental and health applications^11,12^, and planning interventions^13^. Nevertheless, for the majority of these countries, and especially for those most severely and disproportionately affected by both natural disaster and infectious disease morbidity, contemporary, spatially detailed, consistent, and open data on population distribution are often unavailable or difficult to obtain.

For these reasons, since the mid-1990s, there has been an increasing effort to create spatially-explicit population datasets by using a range of approaches, assumptions, and input data to disaggregate administrative unit-based population counts to a regular grid of fixed spatial resolution^14^. Current global gridded datasets depicting the distribution of human population across the Latin America and the Caribbean region include various versions of the Gridded Population of the World (GPW)^15–18^, the Global Rural-Urban Mapping Project (GRUMP)^19^, the Oak Ridge National Laboratory's LandScan^20^, and the United Nation Environment Programme Latin American and Caribbean Population Database^21^. However, these datasets present certain limitations due to their spatial resolution ranging between 30 and 150 arc seconds (approximately 1 and 5 km at the equator, respectively), the age and coarse spatial detail of the input population count data, or the lack of details on the input data and modelling approach used to produce them^22,23^.

**Figure 1:**
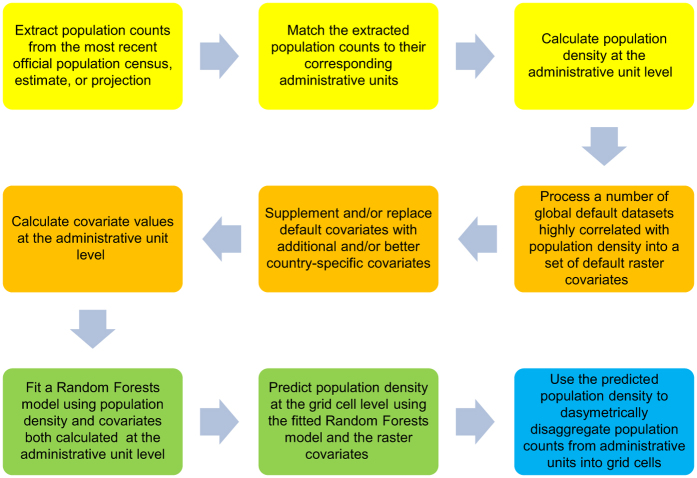
Schematic overview of the Random Forest (RF)-based dasymetric mapping approach used to produce the WorldPop Americas datasets (modified from Stevens *et al.*^24^). The preparation of the response variable and covariates is described in the yellow and orange panels, respectively, the RF modelling steps are outlined in in the green panels, and the disaggregation of the input population counts from administrative units into grid cells is described in the blue panel.

In the framework of the WorldPop Project (www.worldpop.org), an open access archive of high-resolution gridded population distribution datasets for the Latin America and the Caribbean region has been created using the most recent and finest level census and official population estimate data available at the time of writing, along with a range of ancillary geospatial datasets depicting factors known to relate to human population presence and densities. Following the Random Forest (RF)-based dasymetric mapping approach developed by Stevens *et al.* ^24^ (Fig. 1), population count data and ancillary datasets for 28 countries (Tables 1 and 2) were identified, collected, assembled, and exploited in order to produce gridded population datasets with a spatial resolution of 3 arc seconds (approximately 100 m at the equator). These datasets were produced for the population count year, as well as for 2010, 2015, and 2020 using the United Nations Population Division (UNPD) rural and urban growth rates^25^; with national totals for 2010, 2015, and 2020 both remaining unadjusted and being adjusted to match UNPD estimates^25^.

## Methods

### Random forest-based dasymetric population mapping approach

The dasymetric disaggregation of population counts from administrative units into grid cells was undertaken using a population density weighting layer generated by a RF algorithm. RF is a non-linear and non-parametric ensemble learning method that generates a large collection of unpruned decision tree models and aggregates their predictions. Each tree is independently generated by bagging (i.e., by bootstrapping with replacement)^26^, and each node of each tree is split using the optimal split among a randomly selected subset of covariates^27^. Outputs of all tree models are then aggregated by calculating either their mode or average, depending on whether the decision trees are used for classification or regression.

The RF method is robust to overfitting^27^ and not very sensitive, in terms of affecting prediction accuracy, to the three parameters required to be set for fitting the model^28^, namely (i) the number of covariates to be randomly selected at each node, (ii) the number of observations in the terminal nodes of each trees, and (iii) the number of trees in the forest. Furthermore, it is possible to accurately estimate the prediction error of the RF model. This can be done by averaging all mean squared errors calculated using the ‘out-of-bag’ (OOB) data that represent one third of the observations withheld from the bagging iteration process for each tree in the forest^27^. The OOB error can be also used to evaluate the importance of each covariate by considering how much the OOB error increases when only the OOB data for that given covariate are permuted^28,29^.

**Figure 2:**
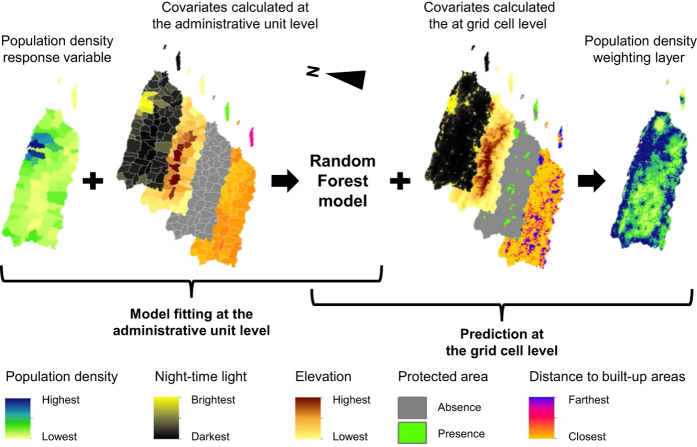
Schematic overview of the procedure used to generate population density weighting layers. For illustrative purpose, only 4 out of the 74 covariates considered for Puerto Rico are shown here (the uninhabited Puerto Rican islands of Mona, Monito, and Desecheo are not shown).

In the RF-based dasymetric population mapping approach developed by Stevens *et al.* ^24^, a RF algorithm is used to generate gridded population density estimates that are subsequently used to dasymetrically disaggregate population counts from administrative units into grid cells. The same approach was used to produce the WorldPop Americas datasets described in this article. Initially, a population density response variable and a suite of covariates were calculated at the administrative unit level, and then used to fit a RF model for predicting population density at the grid cell level (i.e., to generate the dasymetric weighting layer) with those raster-based covariates having a spatial resolution of 100 m (Fig. 2).

To reduce processing time during the prediction phase, the multi-stage RF estimation technique developed by Stevens *et al.* ^24^ was used. This technique first fits a model using all available covariates and the (log) population density of each administrative unit as the response. Then, a very conservative covariate selection process is performed to reduce the number of covariates that will be used for both the RF model fitting and prediction. To do this the ‘variable’ importance of each covariate^27^ is extracted and each covariate that has a score equal to zero is removed before re-fitting the RF model. This process is then iterated until only covariates with positive scores remain and thus results in the elimination of both redundant covariates and covariates that could negatively impact the prediction.

As in Stevens *et al.*^24^, the RF model fitting was performed by generating 500 trees in the forest and setting the number of observations in the terminal nodes equal to one. The fitted RF model was then used to predict population density using only the same reduced set of covariates. For each grid cell, each regression tree in the forest was used to predict a population density value and the average of all predictions was assigned to it as its estimated population density value. If there were not enough observations (i.e., not enough administrative unit population counts) to fit a RF model for a given country, another country located in the same ecozone^30^ was identified and used to fit an appropriate RF model for predicting population density at the grid cell level^31^.

**Figure 3:**
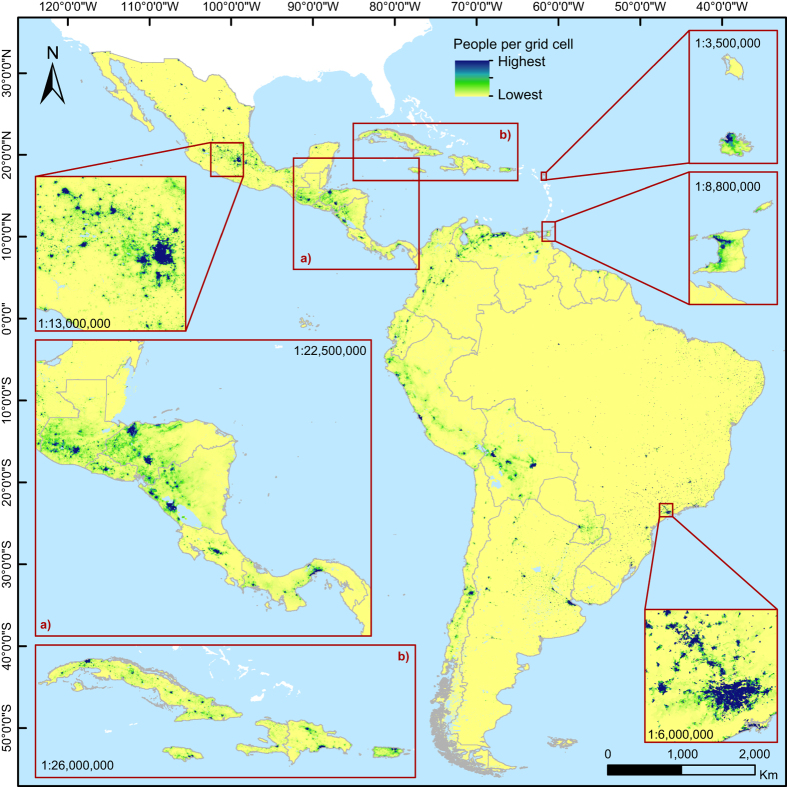
Estimated people per grid cell for Latin America and the Caribbean in 2010 (excluding Guadalupe, Martinique, Bahamas, Barbados, Saint Lucia, Curaçao, Aruba, Saint Vincent and The Grenadines, US and British Virgin Islands, Grenada, Dominica, Cayman Islands, Saint Kitts and Nevis, Sint Maarten, Turks and Caicos Islands, Saint Martin, Caribbean Netherlands, Anguilla, Saint Barthélemy, and Montserrat). The grid cell resolution is 3 arc seconds (approximately 100 m at the equator) and coordinates refer to GCS WGS 1984. For illustrative purpose, the color ranges used are country-specific.

Subsequently, in both cases, the population density weighting layer was used to dasymetrically disaggregate the administrative unit-based population counts^32^ and produce two gridded population datasets depicting the estimated number of people per grid square and per hectare for the population count year. These datasets were then projected to 2010 (Fig. 3), 2015, and 2020 using UNPD rural and urban growth rates^25^ and also adjusted to match the most recent UNPD estimates at the time of writing^25^.

All tasks described above were entirely performed using the WordPop-RF code (Data Citation 1) described in the Code availability section below and publicly available through the *figshare* repository. In particular, the code relies on the R statistical environment (version 2.15) and the randomForest package (version 4.6–7) for fitting the RF model at the administrative unit level and predict at the grid cell level, and on the Python programming language (version 2.6; https://www.python.org/) and ArcGIS 10.1 arcpy package for performing the Geographic Information System (GIS)-specific spatial operations required for dasymetrically disaggregating the population data, projecting them to 2010, 2015 and 2020, and adjusting them to match UNPD estimates (refer to the Supplementary File 1 for a technical description of how the GIS-specific spatial operations are implemented).

### Data collection

For each country listed in Table 1, population counts were extracted from the most detailed and recent official population count data and matched to their corresponding administrative units in a GIS environment. Both population counts and the corresponding administrative units were either publicly available (e.g., from GeoHive^33^ and GADM^34^, respectively) or contributed by National Statistical Offices such as the Instituto Brasileiro de Geografia e Estatística (IBGE). Table 1 also provides summary information about the input population count data and administrative unit datasets used to produce the WorldPop Americas datasets.

It is well known that human population density is highly correlated with environmental and physical factors^35^ that can plausibly impact the spatial distribution of population and/or be related to it. These may include continuous variables such as intensity of night-time lights^36^, energy productivity of plants^37^, topographic elevation and slope^38,39^, and climatic factors^40^, as well as categorical variables such as land-cover type^41^ and presence/absence of roads^42^, waterways and waterbodies^43^, human settlements and urban areas^44^, and protected areas^45^. Thus, twelve global raster and vector datasets (described below) were identified, collected, assembled, and processed into a set of default covariates (Table 2) used for model fitting and prediction.

The spatial variation of factors related to population distribution, such as night-light intensity and plant energy productivity, was measured using the NOAA Suomi National Polar-orbiting Partnership Visible Infrared Imaging Radiometer Suite (VIIRS)^46,47^ and the NASA TERRA/Moderate Resolution Imaging Spectroradiometer (MODIS) Net Primary Productivity (NPP)^48,49^ raster dataset, respectively. The spatial variation of climatic factors affecting population distribution was considered by including the WorldClim Annual Mean Temperature (BIO_1_) and Annual Precipitation (BIO_12_) raster datasets^50,51^. The World Wildlife Fund (WWF) HydroSheds raster dataset^52,53^, based on the NASA’s Shuttle Radar Topography Mission (SRTM) Digital Elevation Model^54^, was used to represent the spatial variation of elevation and slope. The European Space Agency (ESA) ENVISAT/MERIS-based GlobCover raster dataset^55,56^, and the MODIS 500-m map of global urban extent^57,58^ were used to identify different land-cover types and distinguish between urban and rural areas. Finally, the World Database on Protected Areas (WDPA)^59^ was used to obtain vector polygons representing protected areas, while the National Geospatial-Intelligence Agency (NGA) Vector Map Level 0 (VMAP0) dataset^60^ was used to obtain features representing populated places, roads, rivers, and waterbodies.

Where available, additional country-specific datasets were used to integrate and/or replace the default datasets outlined above and the corresponding default covariates in the analysis. For example, for most of the countries, the Landsat TM-based EarthSat GeoCover-LC raster dataset^61,62^ was combined with the GlobCover raster dataset to refine the extent of urban areas and identify rural settlements. Similarly, OpenStreetMap (OSM) vector datasets^63^ were regularly used to integrate the VMAP0 settlement dataset and account for land-use types, building sites, and locations of points of interest that may be strongly correlated with population presence (e.g., health clinics, schools, and police stations). Furthermore, OSM road and river data were often deemed to be more complete than the corresponding VMAP0 data and, thus, were used to increase the precision and accuracy of the gridded population outputs^64^.

For each country, all assembled vector and raster datasets, including the country specific ones, are described in the metadata file accompanying the corresponding gridded population datasets and viewable in any web-browser (refer to the Data Record section below for a more detailed description of the metadata file content).

### Data preparation

For each country, the vector dataset representing its administrative units, used to match to population counts, was projected using the most appropriate country-specific projected coordinate system that minimized linear and areal distortion. It was then buffered by 10 km, and rasterized at a spatial resolution of 100 m. This was done in order to (i) generate a dataset representing the population density response variable, (ii) obtain a raster dataset, representing the study area, for co-registering all raster covariates, and (iii) produce a number of raster ‘distance to’ covariates that were unaffected by edge effects due to the fact that the study area is artificially bounded while spatial processes are not^65^. The population density response variable was obtained through dividing population counts by the area of the corresponding administrative units, and log-transforming the results to normalize the response variable distribution.

Covariates for input to the RF method were derived as follows. First, a continuous raster dataset representing the spatial variation of topographic slope was derived from the USGS HydroSheds dataset (Table 2). Then, all raster datasets representing continuous variables, including the latter, were projected, resampled to 100 m resolution, co-registered and matched to the rasterized buffered study area. For all covariates, ‘NoData’ grid cells overlapping the rasterized buffered study area were filled with the values of the nearest neighbours (using the Nibble tool available in ArcGIS 10.1). All vector and raster datasets representing categorical variables were projected, rasterized to or resampled to 100 m resolution, co-registered, matched to the rasterized buffered study area, and converted into a number of binary raster covariates, representing presence/absence of a given feature, that were subsequently used to produce continuous ‘distance to’ and ‘proportion of’ raster covariates (Table 2); with the latter representing, within a 500 m buffer from each grid cell, the proportion of grid cells where the given feature is present.

A special case of a categorical raster dataset is the land-cover data. Indeed, in this case land-cover classes must be aggregated (if needed) and recoded to match the ten WorlPop Americas classes derived from the GlobCover dataset (i.e., from class 11 to 230 in the 4th column of Supplementary Table 1). By default, the recoded GlobCover dataset was ‘Nibbled’, to fill in any missing grid cell, and then mosaicked with the MODIS 500 m Global Urban Extent dataset to delineate the extent of urban and non-urban built-up areas (i.e., class 190 and 240 respectively in Supplementary Table 1). When using the GeoCover-LC dataset, it was first recoded and mosaicked with the GlobCover dataset, to fill missing grid cell, and with the MODIS 500 m Global Urban Extent dataset. Similarly to the other raster datasets representing categorical variables, the processed land-cover raster dataset obtained as described above was projected, resampled to 100 m resolution, co-registered, matched to the rasterized buffered study area, and converted into twelve binary raster covariates including the combined built-up areas class (BLT) obtained by combining classes 190 and 240. Binary raster covariates were subsequently used to produce continuous ‘distance to’ and ‘proportion of’ raster covariates (Table 2). Finally, average and modal values for continuous and binary covariates, respectively, were calculated for each administrative unit and used for fitting the RF model.

The preparation of the population density response variable and raster covariates was entirely performed using the WordPop-RF code (Data Citation 1) described in the Code availability section below and publicly available through the *figshare* repository. In particular, the code relies on the Python programming language (version 2.6; https://www.python.org/) and ArcGIS 10.1 arcpy package for performing the GIS-specific spatial operations required for preparing both the response variable and raster covariates (refer to the Supplementary File 1 for a technical description of how the GIS-specific spatial operations are implemented). For each country, all derived covariates are listed in the metadata file accompanying the corresponding gridded population datasets (refer to the Data Record section below for a more detailed description of the metadata file contents).

### Code availability

The WordPop-RF code (Data Citation 1), used to produce the WorldPop Americas datasets, as well as the metadata and the KML files associated with them (refer to the Data Records section for a description of the latter), is publicly available through the *figshare* repository. The code consists of two Python (version 2.6; https://www.python.org/) and four R (version 2.15.3) programming language scripts that must be run sequentially in the following order: 1) 01.0—Configuration.py.R; 2) Metadata.R; 3) 01.1—Data Preparation, R.r; 4) 01.2—Data Preparation, Python.py; 5) 01.3—More Complex Random Forest Regression, Full Covariate Set and Data Preparation.r; 6) 01.4—Process Density Weights to Population Maps.py; 7) 01.5—Generate KML.r; 8) 01.6—Generate Metadata Report.r. Each script is also internally documented in order to both explaining its purpose (including a detailed description of the GIS-specific spatial operations that it performs) and, when required, guiding the user through its customization.

## Data Records

The high-resolution WorldPop Americas datasets described in this article referring to the 28 countries listed in Table 1, are publicly and freely available both through the WorldPop Dataverse Repository (Data Citation 2) and the WorldPop project website (http://www.worldpop.org.uk/data/). However, while the WorldPop Americas datasets stored in the Dataverse Repository represent a static version of the datasets produced at the time of writing and will be preserved stably in their published form, the datasets stored in the project website (Supplementary Table 2) will be expanded by including additional countries located in the region and updated as better and more recent official population count data and covariates become available.

Both through the Dataverse Repository and the project website, the WorldPop Americas can be download as 7-Zip archives (7-Zip.org) containing the population distribution datasets of the country it is associated with for the population count year, as well as for 2010, 2015, and 2020, and a RF model metadata report (Table 3).

Additionally, from the Data Availability page available on the WorldPop project website (http://www.worldpop.org.uk/data/data_sources/) it is also possible to browse the 7-Zip archives described above, download individual GeoTIFF datasets from them, and view the HTML files containing the RF model metadata reports. For each country, the metadata report illustrates the datasets and the related derived covariates used as input in the RF model, the population density response variable, the gridded population density dataset used to dasymetrically disaggregate the population from administrative unit to grid cell level, and basic information about the RF model that includes (i) the country on which it is based, (ii) its prediction error, (iii) the relative importance of each covariate, (iv) the prediction intervals using the OOB data (refer to the Methods section for additional information about the latter features).

## Technical Validation

### Root mean square error (RMSE) and mean absolute error (MAE)

Six countries, located in different parts of the Latin American and the Caribbean region were selected to assess the increased accuracy of the RF-based dasymetric mapping approach with respect to a simple areal-weighting (SAW) approach^66^ (Table 4). For each selected country, population counts were aggregated within the next coarser administrative level boundary than the finest for which they were available (e.g., if admin level 4 population count data were available, these were aggregated to admin level 3). The coarser, aggregated population counts were then used to produce gridded population count datasets, with a resolution of 100 m, using both the SAW and the RF approach outlined here. Finally, the two different population estimates produced using these approaches within each of the finest administrative unit were calculated, and compared with observed population figure referring to the same higher resolution unit.

Results, summarized in Table 4, show how both the RMSE, the %RMSE (RMSE expressed as a percentage of the average population of the finest administrative unit level), and the MAE values (5th, 6th, and 7th column of Table 4, respectively) calculated using the RF-based outputs are consistently lower than the corresponding values calculated for the SAW outputs. These statistics can be used to compare the accuracy of the two approaches when downscaling the estimates.

### Out-of-bag (OOB) error estimation

The OOB error estimate (3rd column of Table 4), as already briefly described in the Methods section, is internally calculated during the RF model fitting and can be considered a robust and unbiased measurement of the prediction accuracy of the model itself^27^.

Nevertheless, it is important to note that since the RF model is fitted at the administrative unit level and then is used to predict at the grid cell level, the OOB error estimate should not be interpreted as the prediction error at the grid cell level. Similarly, it does not represent the prediction error that could be expected to be observed at the administrative unit level by summing all final grid cell values within each administrative unit and comparing it to the observed population count referring to the same administrative unit. However, referring to the six countries mentioned in the previous section, by comparing the OOB error estimates calculated at the aggregate lower administrative unit level than the highest available (3rd column of Table 4) with the corresponding RMSE and MAE values (5th and 7th column of Table 3, respectively), it is reasonable to expect that higher accuracy of predicted values at the administrative unit level results in a higher accuracy of the final gridded population distribution datasets^24^.

## Usage Notes

The WorldPop Americas datasets can be used both to support applications for planning interventions, measuring progress, and to predict response variables intrinsically dependent on the population distribution. However, considering that they represent modelling outputs generated using ancillary covariate datasets in the disaggregation process, to avoid circularity, they should not be used to make predictions or explore relationships about any of these ancillary datasets^14^. Thus, before using WorldPop Americas datasets in correlation analyses against factors which are included in the process of their construction (e.g., correlating population distribution with land-cover), ideally the population modelling process should be re-run using the WordPop-RF code (Data Citation 1) with the covariate of interest being removed to avoid issues relating to endogeneity.

## Additional Information

**How to cite this article:** Sorichetta, A. *et al.* High-resolution gridded population datasets for Latin America and the Caribbean in 2010, 2015, and 2020. *Sci. Data* 2:150045 doi: 10.1038/sdata.2015.45 (2015).

## Supplementary Material



Supplementary Table 1

Supplementary Table 2

Supplementary File 1

## Figures and Tables

**Table 1:** Summary information about population count data and administrative unit datasets used to produce the WorldPop Americas datasets

**ISO code**	**Area (sqkm)**	**Total population**	**Year**	**No. of units**	**Unit name/level**	**Average spatial resolution**	**Population data source**	**Units data source**
ATG	436	81,799^C^	2011	8	Parish/1	2.6	Census Office^G^	GADM^34^
ARG	2,804,771	40,117,096^C^	2010	526	Department/2	3.2	INDEC^G^	IGN^67^
BLZ	21,918	312,971^C^	2010	16	Subdivision/1	9.3	Statistical Institute of Belize^G^	Meerman^68^
BOL	1,069,327	10,027,262^C^	2012	112	Province/2	9.2	INE^G^	GADM^34^
BRA	8,233,131	190,732,694^C^	2010	5565	Municipality/3	0.6	IBGE	IBGE
CHL	756,096	16,341,929^C^	2012	297	Municipality/3	2.9	INE-CELADE^G^	GADM^34^
COL	1,141,261	47,661,787^E^	2013	1115	Municipality/2	1.0	Departamento Administrativo Nacional de Estadística^G^	GADM^34^
CRI	51,100	4,301,712^C^	2011	469	District/3	0.5	INEC^G^	GADM^34^
CUB	109,884	11,167,325^C^	2012	168	Municipality/2	2.0	ONE^G^	GADM^34^
DOM	48,070	9,445,281^C^	2010	155	Municipality/3	1.4	ONE^G^	GADM^34^
ECU	257,320	14,483,499^C^	2010	978	Parish/4	0.5	INEC^G^	GADM^34^
SLV	21,045	5,744,113^C^	2007	267	Municipality/2	0.5	Dirección General de Estadística y Censos^G^	GADM^34^
GUF	83,534	231,167^E^	2010	21	Municipality/3	13.8	Insee^G^	GADM^34^
GTM	108,201	15,073,375^P^	2012	335	Municipality/2	1.0	INE^G^	GADM^34^
GUY	214,999	751,223^C^	2002	116	Council/2	4.0	Statistics Guyana^G^	GADM^34^
HTI	26,964	9,923,243^E^	2009	570	Section/4	0.3	IHSI	GADM^34^
HND	112,457	8,045,990^P^	2010	298	Municipality/2	1.1	INE^G^	GADM^34^
JAM	10,991	2,697,983^C^	2011	14	Parish/1	7.5	Statistical Institute^G^	GADM^34^
MEX	19,67,138	112,336,538^C^	2010	2456	Municipality/2	0.6	INEGI	Valle-Jones^69^
NIC	120,340	6,071,045^E^	2012	139	Municipality/3	2.5	INIDE^G^	GADM^34^
PAN	741,77	3,405,813^C^	2010	77	District/2	3.5	Dirección de Estadística y Censo^G^	GADM^34^
PRY	406,752	3,725,789^E^	2002	247	Municipality/2	2.6	Dirección General de Estadística, Encuestas y Censos^G^	GADM^34^
PER	1,294,681	30,135,875^P^	2012	194	Province/2	5.9	INEI^G^	GADM^34^
PRI	13,790	3,725,789^C^	2010	78	Municipality/1	1.5	U.S. Census Bureau^G^	GADM^34^
SUR	163,820	541,638^C^	2004	62	Resort/2	6.5	Algemeen Bureau voor de Statistiek^G^	GADM^34^
TTO	5127	1,328,019^C^	2011	14	Municipality/1	5.1	Central Statistical Office^G^	GADM^34^
URY	175,016	3,286,314^C^	2011	19	Department/1	22.0	INE^G^	GADM^34^
VEN	913,982	28,946,101^C^	2011	344	Municipality/2	2.8	INE^G^	GADM^34^
For each country (identified by its ISO country code in the 1st column), the Average Spatial Resolution was calculated as the square root of its surface area divided by the number of administrative units and represents the effective resolution of the latter (i.e., the cell size of administrative units if all units were square of equal size)^14^. Superscripts ‘C’, ‘E’, and ‘P’, in the 2nd column, indicate whether the population counts were obtained from either official census, estimates, or projections, respectively. Superscript ‘G’, in the 8th column, indicates that the population counts were downloaded from GeoHive^33^.								

**Table 2:** Summary information on the twelve default datasets and the derived default covariates used for input to the RF method

**Default dataset**	**Default derived covariate**	**Temporal coverage**	**Type**	**Format**	**Resolution**	**Source**
**Suomi NPP-VIIRS**		**2012**	**Continuous**	**Raster**	**15 arc seconds**	**NOAA**^46^
	Night-lights’ intensity	2012	Continuous	Raster	100 m	
**MODIS Net Primary Production**		**2014/2015**	**Continuous**	**Raster**	**30 arc seconds**	**NASA**^48^
	Plants’ energy productivity	2014/2015	Continuous	Raster	100 m	
**WorldClim (BIO**_**1**_)		**1950–2000**	**Continuous**	**Raster**	**30 arc seconds**	**Hijmans** ***et al.***^50^
	Annual Mean Temperature	1950–2000	Continuous	Raster	100 m	
**WorldClim (BIO**_**12**_)		**1950–2000**	**Continuous**	**Raster**	**30 arc seconds**	**Hijmans** ***et al.***^50^.
	Annual Precipitation	1950–2000	Continuous	Raster	100 m	
**HydroSheds (3 s GRID: Void-filled DEM)**		**2000**	**Continuous**	**Raster**	**3 arc seconds**	**WWF**^52^
	Elevation	2000	Continuous	Raster	100 m	
	Slope	2000	Continuous	Raster	100 m	
**MERIS GlobCover**		**2009**	**Categorical**	**Raster**	**10 arc seconds**	**ESA**^55^
	Presence/absence of class #	2000/2009	Categorical (binary)	Raster	100 m	
	Distance to class #	2000/2009	Continuous	Raster	100 m	
	Proportion of class #	2000/2009	Continuous	Raster	100 m	
	Presence/absence of built-up areas (BLT)	2000/2009	Categorical (binary)	Raster	100 m	
	Distance to built-up areas (BLT)	2000/2009	Continuous	Raster	100 m	
	Proportion of built-up area (BLT)					
**MODIS 500 m Global Urban Extent**		**2000/2001**	**Categorical (binary)**	**Raster**	**15 arc seconds**	**Schneider** ***et al.***^57^
	Presence/absence of urban areas	2000/2001	Categorical (binary)	Raster	100 m	
	Distance to urban areas	2000/2001	Continuous	Raster	100 m	
	Proportion of urban area	2000/2001	Continuous	Raster	100 m	
**World Database on Protected Areas**		**2012**	**Categorical**	**Vector**	**—**	**UNEP-WCMC & IUCN**^59^
	Presence/absence of protected areas	2012	Categorical (binary)	Raster	100 m	
	Distance to protected areas	2012	Continuous	Raster	100 m	
	Proportion of protected area	2012	Continuous	Raster	100 m	
**VMAP0 populated places/roads/rivers/waterbodies**		**1979–1999**	**Categorical**	**Vector**	**—**	**NGA**^60^
	Presence/absence of populated places/roads/rivers/waterbodies		Categorical (binary)	Raster	100 m	
	Distance to populated places/roads/rivers/waterbodies		Continuous	Raster	100 m	
	Proportion of populated places/roads/rivers/waterbodies		Continuous	Raster	100 m	
Continuous raster datasets were resampled for being used as covariates, while both categorical raster and rasterized vector data sets were firstly resampled and then processed into ‘presence/absence’, ‘distance to’, and ‘proportion of’ raster covariates. ‘Class #’, in the 2nd column, refers to the WorlPop Americas classes described in Supplementary Table 1. Refer to the Data preparation sub-section below for a more detailed description of how default covariates were processed.						

**Table 3:** Name (ISO and YEAR represent the ISO country code and the population count year, respectively), description, and format of all files contained in each 7-Zip archive associated with the 28 countries listed in Table 1.

**Name**	**Description (resolution)**	**Format**
ISO_ppp_v2b_YEAR.tif	Estimated people per grid cell for the year the official population count data refer to (3 arc seconds)	GeoTIFF
ISO_ppp_v2b_2010.tif	Projected estimated people per grid cell for 2010 (3 arc seconds)	GeoTIFF
ISO_ppp_v2b_2010_UNadj.tif	Projected estimated people per grid cell for 2010 adjusted to match UNPD estimates (3 arc seconds)	GeoTIFF
ISO_ppp_v2b_2015.tif	Projected estimated people per grid cell for 2015 (3 arc seconds)	GeoTIFF
ISO_ppp_v2b_2015_UNadj.tif	Projected estimated people per grid cell for 2015 adjusted to match UNPD estimates (3 arc seconds)	GeoTIFF
ISO_ppp_v2b_2020.tif	Projected estimated people per grid cell for 2020 (3 arc seconds)	GeoTIFF
ISO_ppp_v2b_2020_UNadj.tif	Projected estimated people per grid cell for 2020 adjusted to match UNPD estimates (3 arc seconds)	GeoTIFF
ISO_pph_v2b_YEAR.tif	Estimated people per hectare for the year the official population count data refer to (3 arc seconds)	GeoTIFF
ISO_pph_v2b_2010.tif	Projected estimated people per hectare for 2010	GeoTIFF
ISO_pph_v2b_2010_UNadj.tif	Projected estimated people per hectare for 2010 adjusted to match UNPD estimates (3 arc seconds)	GeoTIFF
ISO_pph_v2b_2015.tif	Projected estimated people per hectare for 2015	GeoTIFF
ISO_pph_v2b_2015_UNadj.tif	Projected estimated people per hectare for 2015 adjusted to match UNPD estimates (3 arc seconds)	GeoTIFF
ISO_pph_v2b_2020.tif	Projected estimated people per hectare for 2020 (3 arc seconds)	GeoTIFF
ISO_pph_v2b_2020_UNadj.tif	Projected estimated people per hectare for 2020 adjusted to match UNPD estimates (3 arc seconds)	GeoTIFF
ISO_ppp_v2b_YEAR.kmz	Estimated people per grid cell for the year the official census/population counts refer to	Keyhole Markup Language (Zipped)
ISO_metadata.html	Metadata report for the Random Forest model	HyperText Markup Language

**Table 4:** Prediction accuracy of the RF model used to generate the dasymetric weighting layers and accuracy assessment of the RF-based dasymetric mapping approach compared to the simple areal-weighting (SAW) mapping approach

**ISO code**	**Model**	**Unit level**	**No. of units**	**OOB error**	**% of variation explained**	**RMSE**	**RMSE%**	**MAE**
ATG	RF	1	8	0.21	86	—	—	—
ARG	RF	2	526	0.78	88	—	—	—
BLZ	RF	1	16	0.25	79	—	—	—
BOL	RF	2	112	0.88	65	—	—	—
BRA	RF	3	5565	0.32	84	—	—	—
CHL	RF	3	297	1.40	70	—	—	—
COL	RF	2	1115	0.35	84	—	—	—
COL	RF	1	33	1.20	75	109798.10	259.81	29361.29
COL	SAW	1	33	—	—	128372.29	303.76	36463.22
CRI	RF	3	469	0.40	92	—	—	—
CRI	RF	2	81	0.20	93	4837.37	52.96	3012.04
CRI	SAW	2	81	—	—	14463.43	158.34	7976.94
CUB	RF	2	168	0.33	82	—	—	—
DOM	RF	2	155	0.22	86	—	—	—
DOM	RF	1	32	0.53	62	46349.33	76.06	19461.99
DOM	SAW	1	32	—	—	101563.30	166.67	39729.54
ECU	RF	4	978	0.47	82	—	—	—
ECU	RF	3	198	0.43	77	36713.59	248.75	7243.05
ECU	SAW	3	198	—	—	60295.60	408.52	12322.64
SLV	RF	2	267	0.20	81	—	—	—
GUF	RF	3	21	2.60	59	—	—	—
GTM	RF	2	335	0.24	80	—	—	—
GTM	RF	1	22	0.33	58	51704.90	114.13	20590.36
GTM	SAW	1	22	—	—	62299.18	137.52	26125.56
GUY	RF	2	116	1.10	87	—	—	—
HTI	RF	4	570	0.14	84	—	—	—
HTI	RF	3	140	0.071	90	10794.96	62.01	5493.25
HTI	SAW	3	140	—	—	18677.50	107.29	8501.97
HND	RF	2	298	0.20	71	—	—	—
JAM	RF	1	14	0.21	86	—	—	—
MEX	RF	2	2456	0.21	92	—	—	—
NIC	RF	3	139	0.32	79	—	—	—
PAN	RF	2	77	0.41	74	—	—	—
PRY	RF	2	247	0.44	85	—	—	—
PER	RF	2	194	0.58	63	—	—	—
PRI	RF	1	78	0.16	74	—	—	—
SUR	RF	2	62	1.40	86	—	—	—
TTO	RF	1	14	0.21	86	—	—	—
URY	RF	1	19	0.58	91	—	—	—
VEN	RF	2	344	1.20	71	—	—	—
The OOB error and the percentage of variance explained are provided for all 28 countries while the RMSE, the %RMSE, and the MAE values are provided for six countries. ‘RF’ and ‘SAW’, in the 2nd column, indicate that, for that specific country, the population counts at the administrative unit level were disaggregated using the RF-based dasymetric mapping approach and the simple areal-weighting approach, respectively.								
