# Supplementary material for: High-resolution gridded population datasets for Latin America and the Caribbean in 2010, 2015, and 2020
Source: Sci Data. 2015 Sep 1;2:150045. doi: 10.1038/sdata.2015.45 (PMC4555876; doi:10.1038/sdata.2015.45)
Supplement: Supplementary Table 1 [file sdata201545-s2.doc]

| **GlobCover class value** | **GlobCover class description** | **WorlPop Americas class description** | **WorlPop Americas class value** |
| --- | --- | --- | --- |
| 11 | Post-flooding or irrigated croplands (or aquatic) | Cultivated terrestrial areas and managed lands | 11 |
| 14 | Rainfed croplands |
| 20 | Mosaic cropland (50-70%) / vegetation (grassland/shrubland/forest) (20-50%) |
| 30 | Mosaic vegetation (grassland/shrubland/forest) (50-70%) / cropland (20-50%) |
| 40 | Closed to open (>15%) broadleaved evergreen or semi-deciduous forest (>5m) | Natural and semi-natural terrestrial vegetation - Woody/Trees | 40 |
| 50 | Closed (>40%) broadleaved deciduous forest (>5m) |
| 60 | Open (15-40%) broadleaved deciduous forest/woodland (>5m) |
| 70 | Closed (>40%) needleleaved evergreen forest (>5m) |
| 90 | Open (15-40%) needleleaved deciduous or evergreen forest (>5m) |
| 100 | Closed to open (>15%) mixed broadleaved and needleleaved forest (>5m) |
| 110 | Mosaic forest or shrubland (50-70%) / grassland (20-50%) |
| 120 | Mosaic grassland (50-70%) / forest or shrubland (20-50%) |
| 130 | Closed to open (>15%) (broadleaved or needleleaved, evergreen or deciduous) shrubland (<5m) | Natural and seminatural terrestrial vegetation - Shrubs | 130 |
| 140 | Closed to open (>15%) herbaceous vegetation (grassland, savannas or lichens/mosses) | Natural and seminatural terrestrial vegetation - Herbaceous | 140 |
| 150 | Sparse (<15%) vegetation | Natural and semi-natural sparse terrestrial vegetation | 150 |
| 160 | Closed to open (>15%) broadleaved forest regularly flooded (semi-permanently or temporarily) - Fresh or brackish water | Natural and seminatural aquatic vegetation | 160 |
| 170 | Closed (>40%) broadleaved forest or shrubland permanently flooded - Saline or brackish water |
| 180 | Closed to open (>15%) grassland or woody vegetation on regularly flooded or waterlogged soil - Fresh, brackish or saline water |
| 190 | Artificial surfaces and associated areas (Urban areas >50%) | Artificial surfaces | 190 |
| 200 | Bare areas | Bare areas | 200 |
| 210 | Water bodies | Inland waterbodies, snow and ice | 210 |
| 220 | Permanent snow and ice |
| 230 | No data (burnt areas, clouds…) | No data (burnt areas, clouds...) | 230 |
| N/A | N/A | Rural settlements | 240 |

Supplementary Table 1. WorlPop Americas land-cover classes derived from the GlobCover dataset (modified form Bontemps *et al.*^49^).
