# Supplementary material for: High-resolution gridded population datasets for Latin America and the Caribbean in 2010, 2015, and 2020
Source: Sci Data. 2015 Sep 1;2:150045. doi: 10.1038/sdata.2015.45 (PMC4555876; doi:10.1038/sdata.2015.45)
Supplement: Supplementary Table 2 [file sdata201545-s3.doc]

| **Country** | **ISO code** | **Dataset URL identifier** |
| --- | --- | --- |
| Antigua and Barbuda | ATG | http://www.worldpop.org.uk/data/summary/?contselect=America&countselect=Antigua%20and%20Barbuda&typeselect=Population |
| Argentina | ARG | http://www.worldpop.org.uk/data/summary/?contselect=America&countselect=Argentina&typeselect=Population |
| Belize | BLZ | http://www.worldpop.org.uk/data/summary/?contselect=America&countselect=Belize&typeselect=Population |
| Bolivia | BOL | http://www.worldpop.org.uk/data/summary/?contselect=America&countselect=Bolivia&typeselect=Population |
| Brazil | BRA | http://www.worldpop.org.uk/data/summary/?contselect=America&countselect=Brazil&typeselect=Population |
| Chile | CHL | http://www.worldpop.org.uk/data/summary/?contselect=America&countselect=Chile&typeselect=Population |
| Colombia | COL | http://www.worldpop.org.uk/data/summary/?contselect=America&countselect=Colombia&typeselect=Population |
| Costa Rica | CRI | http://www.worldpop.org.uk/data/summary/?contselect=America&countselect=Costa%20Rica&typeselect=Population |
| Cuba | CUB | http://www.worldpop.org.uk/data/summary/?contselect=America&countselect=Cuba&typeselect=Population |
| Dominican Republic | DOM | http://www.worldpop.org.uk/data/summary/?contselect=America&countselect=Dominican%20Republic&typeselect=Population |
| Ecuador | ECU | http://www.worldpop.org.uk/data/summary/?contselect=America&countselect=Ecuador&typeselect=Population |
| El Salvador | SLV | http://www.worldpop.org.uk/data/summary/?contselect=America&countselect=El%20Salvador&typeselect=Population |
| French Guiana | GUF | http://www.worldpop.org.uk/data/summary/?contselect=America&countselect=French%20Guiana&typeselect=Population |
| Guatemala | GTM | http://www.worldpop.org.uk/data/summary/?contselect=America&countselect=Guatemala&typeselect=Population |
| Guyana | GUY | http://www.worldpop.org.uk/data/summary/?contselect=America&countselect=Guyana&typeselect=Population |
| Haiti | HTI | http://www.worldpop.org.uk/data/summary/?contselect=America&countselect=Haiti&typeselect=Population |
| Honduras | HND | http://www.worldpop.org.uk/data/summary/?contselect=America&countselect=Honduras&typeselect=Population |
| Jamaica | JAM | http://www.worldpop.org.uk/data/summary/?contselect=America&countselect=Jamaica&typeselect=Population |
| Mexico | MEX | http://www.worldpop.org.uk/data/summary/?contselect=America&countselect=Mexico&typeselect=Population |
| Nicaragua | NIC | http://www.worldpop.org.uk/data/summary/?contselect=America&countselect=Nicaragua&typeselect=Population |
| Panama | PAN | http://www.worldpop.org.uk/data/summary/?contselect=America&countselect=Panama&typeselect=Population |
| Paraguay | PRY | http://www.worldpop.org.uk/data/summary/?contselect=America&countselect=Paraguay&typeselect=Population |
| Peru | PER | http://www.worldpop.org.uk/data/summary/?contselect=America&countselect=Peru&typeselect=Population |
| Puerto Rico | PRI | http://www.worldpop.org.uk/data/summary/?contselect=America&countselect=Puerto%20Rico&typeselect=Population |
| Suriname | SUR | http://www.worldpop.org.uk/data/summary/?contselect=America&countselect=Suriname&typeselect=Population |
| Trinidad and Tobago | TTO | http://www.worldpop.org.uk/data/summary/?contselect=America&countselect=Trinidad-Tobago&typeselect=Population |
| Uruguay | URY | http://www.worldpop.org.uk/data/summary/?contselect=America&countselect=Uruguay&typeselect=Population |
| Venezuela | VEN | http://www.worldpop.org.uk/data/summary/?contselect=America&countselect=Venezuela&typeselect=Population |

Supplementary Table 2. URL identifiers for each single WorlPop Americas dataset available through the WorldPop Project website ([www.worldpop.org](http://www.worldpop.org/)).
