# Supplementary material for: High-resolution gridded population datasets for Latin America and the Caribbean in 2010, 2015, and 2020
Source: Sci Data. 2015 Sep 1;2:150045. doi: 10.1038/sdata.2015.45 (PMC4555876; doi:10.1038/sdata.2015.45)
Supplement: Supplementary File 1 [file sdata201545-s4.doc]

**High-resolution gridded population datasets for Latin America and the Caribbean in 2010, 2015, and 2020**

**Alessandro Sorichetta^1,2^, Graeme M. Hornby^3^, Forrest R. Stevens^4^, Andrea E. Gaughan^4^, Catherine Linard^5,6^ and Andrew J. Tatem^1,7,8^**

**Affiliations**

1. Geography and Environment, University of Southampton, UK

2. Institute for Life Sciences, University of Southampton, UK

3. GeoData Institute, University of Southampton, UK

4. Department of Geography and Geosciences, University of Louisville, KY, USA

5. Université Libre de Bruxelles, Brussels, Belgium

6. Fonds National de la Recherche Scientifique, Brussels, Belgium

7. Fogarty International Center, National Institutes of Health, Bethesda, USA

8. Flowminder Foundation, Stockholm, Sweden

Corresponding author: Alessandro Sorichetta ([A.Sorichetta@soton.ac.uk](mailto:A.Sorichetta@soton.ac.uk))

**Supplementary Information**

This Supplementary File describes in detail the Geographic Information System-specific spatial operations performed in ArcGIS 10.1 for (i) preparing the observed population density data and raster covariates as input to the RF modelling methodology and (ii) dasymetrically disaggregating the administrative unit-based population counts to a regular grid of fixed spatial resolution, estimating the latter for 2010, 2015 and 2020 based on UNPD population growth rates, and adjusting the population counts to match United Nations Population Division (UNPD) estimates^1^. All necessary spatial operations are performed using the *arcpy* geoprocessing interface to ArcGIS 10.1 and run using Python 2.7.

**Response variable and raster covariates preparation**

These operations are contained in the "01.2 - Data Preparation, Python.py" file as archived from the publically available code repository [*Data Citation 1*]. In brief, the Python script reads through a series of data folders and converts individual covariate datasets to a series of rasters performing the following spatial operations:

- the population count polygon feature and all feature datasets, having coordinates in geographic coordinate system (GCS WGS84), are projected to the most appropriate country-specific projection chosen to preserve distances, for distance-to calculations, as well as be mostly conformal to minimize other types of distortion upon further data processing;
- the area of each administrative unit (polygon) in the projected population count polygon feature is calculated using the *Calculate Areas* function;
- the projected population count polygon feature is into a point feature (with each points located inside the corresponding polygons) using the *Feature To Point* function (for faster zonal statistics extraction in *R*);
- the projected population count polygon feature is rasterized using the *Feature To Raster* function, selecting the field containing the unique identifiers for each administrative unit to assign values to the output zonal ID raster, and setting the *Output Cell Size* parameters to 100m (thus identifying the country administrative units based on their unique identifiers, to be used for calculation of zonal stats in *R*);
- the projected population count polygon feature is buffered by 10km using the *Buffer* *Analysis* function and setting the *Side Type*, *End Type*, and *Dissolve Type* parameters to *FULL*, *ROUND*, and *ALL*, respectively;
- the *Processing Extent* parameter is set to the extent of the buffered population count polygon feature and all raster operations are snapped to the reprojected land-cover raster (with the latter described below), creating uniform extents and dimensions for all subsequent analyses;
- all projected feature datasets are clipped to the extent of the buffered population count polygon feature using the *Clip* *Analysis* function and rasterized using the *Feature To Raster* function, the latter selecting the *FID* field to assign the values to the output raster, and setting the *Output Cell Size* parameters to 100m;
- the rasterized feature datasets are used to derive rasters representing “*distance to”*, “*presence/absence*”, and “*proportion of”* using, respectively:
  - the *Euclidean Distance* function;
  - the following raster algebra expression to convert all *NoData* pixels to *0* (indicating absence) and all *FID* pixels to *1* (indicating presence): *IsNull(Raster) == 0* where *raster* represents a rasterized feature dataset;
  - the *Focal Statistics* function on the binary *“presence/absence”* raster, produced in the previous step, to calculate the *MEAN* within a circular neighborhood having a radius of ~500m (without considering the *NoData* pixels located within it);
- categorical rasters (such as the one representing land-cover), having coordinates in geographic coordinate system (GCS WGS84), are projected to the chosen country-specific projection using the *Project Raster* function, setting the *Output Cell Size* and the *Resampling Technique* parameters to *100m* and *NEAREST*, respectively;
- the projected categorical rasters are then used to derive binary rasters representing *“presence/absence”* of categories of interest using the following raster algebra expression: *raster==N* where *N* is the pixel value representing the raster class/category of interest (refer to Supplementary Table 1 for the land-cover classes used to produce the “Americas Datasets” [*Data Citation 2*]);
- additionally, the projected land-cover raster is also used to derive a raster representing *“presence/absence”* of built-up area using the following raster algebra expression: *(raster==190)+(raster==240)* where *raster* represents the projected land-cover raster and *190* and *240* represent urban and non-urban built-up area pixel, respectively;
- all *“presence/absence”* rasters produced in the previous two steps are then used to derive rasters representing *“distance to”* and *“proportion of”* using, respectively:
  - the *Euclidean Distance* function – after converting to *NoData* all pixels which value is *0,* by running the following raster algebra expression: *SetNull(raster != 1, 1)* where *raster* represents any *“presence/absence”* raster produced in the previous two steps;
  - the *Focal Statistics* function to calculate the *MEAN* within a circular neighborhood having a radius of ~500m (without considering the NoData pixels located within it);
- continuous rasters, having coordinates in geographic coordinate system (GCS WGS84), are projected to the chosen country-specific projection using the *Project Raster* function, setting the *Output Cell Size* and the *Resampling Technique* parameter to *100m* and *BILINEAR*, respectively;
- additionally, the projected Digital Elevation Model raster is used to derive a *slope* raster using the *Slope* function.

The projected census data and its derivations as well as all derived rasters are then used as input to the “01.3 - More Complex Random Forest Regression, Full Covariate Set and Data Preparation.r” scripts.

**Population raster datasets production**

These operations are contained in the "01.4 - Process Density Weights to Population Maps.py" file as archived from the publically available code repository [*Data Citation 1*]. To dasymetrically disaggregate the population counts from vector-based administrative units into raster grid cells, weighted by predicted population densities from the Random Forest algorithm, the following spatial operations are performed:

- the *Processing Extent* parameter is set to the extent of the population count polygon feature having coordinates in geographic coordinate system (GCS WGS84);
- the population density weighting raster, generated by the Random Forest algorithm, is re-projected to GCS WGS84 using the *Project Raster* function using an *Output Cell Size* and *Resampling Technique* set to *0.000833333 decimal degrees* and *BILINEAR*, respectively;
- the *Processing Snap* parameter is set to the GCS WGS84 population density weighting raster;
- the population count polygon feature is rasterized using the *Feature To Raster* function, selecting the field containing the total population for each administrative unit to assign the values to the output raster, and setting the *Output Cell Size* parameters to *0.000833333 decimal degrees*;
- the predicted population densities from the Random Forest algorithm are summed within each administrative unit using the *Zonal Statistics* function, selecting the GCS WGS84 rasterized administrative unit ID as the dataset to define the zones and the CGS WGS84 population density weighting raster as the raster containing the values to be summed;
- the dasymetrically disaggregated population raster, depicting the number of people per pixel, is then produced by dividing the population density predicted within each grid cell by the total from the *Zonal Statistics* above and multiplying this proportion by the administrative unit total population (i.e., the rasterized population count polygon feature) to attain the disaggregated administrative unit population per each pixel and for each unit within the country.

Then, to produce population rasters for 2010, 2015 and 2020, and adjust the national totals to the UNPD estimates^1^ the following spatial operations are performed:

- first, the projected *land-cover* raster and *“presence/absence” urban* raster (with the latter representing presence and absence of urban areas, indicated by a pixel value equal to 1 and 0, respectively) are re-projected to GCS WGS84 using the *Project Raster* tool and setting the *Output Cell Size* and *Resampling Technique* parameters to 0.000833333 decimal degrees and *NEAREST*, respectively;
- second, the two rasters generated above are combined using the following raster algebra expression: *landcover*Con(urban==1,0,1)+190*(urban)* where *landcover* is the re-projected *land-cover* raster, *urban* is the re-projected *“presence/absence” urban* raster, *1* and *0* identify urban and rural pixels in *urban*, and *190* identify urban built-up pixels in *land-cover;*
- then population rasters for 2010, 2015 and 2020 are generated using the following raster algebra expression: *ppp*(land_urb!=190)*GR_rur+ppp*(land_urb==190)*GR_urb* where *ppp* is the dasymetrically disaggregated population raster depicting the number of people per pixel, *land_urb* is the raster generated in the previous step (with *190* identifying urban pixels), and *GR_rur* and *GR_urb* are the growth rates in rural and urban areas, respectively.
- a total estimated population count raster for each of the years 2010, 2015 and 2020 is then calculated for the entire country using the *Zonal Statistics* function selecting GCS WGS84 population count polygon feature as the dataset to define the zone (i.e., the ISO country code field) and the population raster for the corresponding year as the raster containing the values to be summed; another constant raster is also created with the UNPD^1^ estimated country total for each year using the following raster algebra expression: *tot_pop_i_*0+UNPOP_i_* where *tot_pop_i_* is the total estimated population raster for the year *i,UNPOP_i_* is the UNDP estimated total country population for the year *i,* and *i* can be either 2010, 2015, or 2020;
- UNPD adjusted population rasters for 2010, 2015 and 2020 are generated using the following raster algebra expression: *pop_i_*(UNPD_pop*_i_ */country_tot_pop_i_)* where *pop_i_* is the estimated population raster for the year *i*, *UNPD_pop_i_* and *country_tot_pop_i_* are, respectively, the constant raster created with the UNPD estimated country total and the country total estimated population count raster both for the year *i*, and *i* can be either 2010, 2015, or 2020.

Last, population per hectare raster datasets are produced by repeating the steps described above using the population density weighting raster and the other required datasets with coordinates in their original, projected coordinate system, dimensions and extent, as produced during the data pre-processing stage. These final people per hectare mapping products are distributed at a projected spatial resolution of 100m.

**References**

1. United Nations, Department of Economic and Social Affairs, Population Division (UNPD). *World Urbanization Prospects: The 2014 Revision, Highlights* (United Nations, New York, 2014).

**Data Citations**

1. Stevens, F. R., Gaughan, A. E., Linard, C., Tatem, A. J., Sorichetta, A., Hornby, G. M., Patel, N. N. & Nieves, J. WorldPop-RF, Version 2b.1.1. *figshare*. <http://dx.doi.org/10.6084/m9.figshare.1491490> (2015).
2. Sorichetta, A., Hornby G. M., Stevens F. R., Gaughan A. E., Linard C. & Tatem A. J. Americas Datasets, V1. *Harvard Dataverse.* <http://dx.doi.org/10.7910/DVN/PUGPVR> (2015).
